# Language performance in children with cerebral palsy: the role of cortical auditory processing beyond motor and cognitive factors

**DOI:** 10.3389/fped.2026.1908510

**Published:** 2026-08-31

**Authors:** Mehmet Aslan, Agit Şimşek

**Affiliations:** 1 Department of Otolaryngology, Faculty of Medicine, Inönü University, Malatya, Türkiye; 2 Department of Speech-Language Pathology, Faculty of Health Sciences, İnönü University, Malatya, Türkiye

**Keywords:** auditory processing, CAEP, cerebral palsy, cortical auditory evoked potentials, language development, N1 latency

## Abstract

**Background:**

Language impairments in children with cerebral palsy (CP) are often attributed to motor limitations; however, the contribution of central auditory processing remains insufficiently understood. Cortical auditory evoked potentials (CAEPs) provide an objective means of assessing auditory cortical processing and may offer insight into the neurophysiological mechanisms underlying language outcomes.

**Objective:**

To examine the relationship between cortical auditory processing and language performance in children with cerebral palsy (CP) and to determine whether electrophysiological measures remain associated with language outcomes after adjustment for motor severity, cognitive status, age, and hearing status.

**Methods:**

This cross-sectional case–control study included 48 children with CP and 48 age- and sex-matched typically developing controls. Language performance was assessed using standardized language measures, and CAEP components (P1, N1, and mismatch negativity [MMN]) were recorded using electroencephalography. Group comparisons, correlation analyses, ANCOVA, and multiple linear regression analyses were performed while accounting for demographic and clinical covariates.

**Results:**

Children with CP demonstrated significantly prolonged CAEP latencies, altered amplitudes, and lower receptive and expressive language scores than controls (all *p* < .001). Within the CP group, N1 latency showed the strongest correlation with language performance (*r* = −0.989, *p* < .001). In the multivariable regression model, N1 latency (*β* = −0.700, *p* < .001), GMFCS level (*β* = −0.299, *p* < .001), cognitive status (*β* = 0.090, *p* = .006), and hearing status (*β* = −0.095, *p* = .002) remained statistically associated with total language scores when entered into the same model, whereas age was not (*p* = .390). The model explained 98.7% of the variance in language performance (R² = .987).

**Conclusions:**

Cortical auditory processing measures remained significantly associated with language performance after adjustment for motor severity, cognitive status, age, and hearing status. These findings support the potential value of CAEP measures as complementary neurophysiological markers for the assessment of language impairment in children with cerebral palsy.

## Introduction

Cerebral palsy (CP) is a non-progressive neurodevelopmental disorder resulting from damage to the developing fetal or infant brain, primarily affecting movement, posture, and motor control. However, CP is not limited to motor impairments; it is frequently accompanied by sensory, cognitive, behavioural, and communication difficulties (1, 2). Among these, speech and language impairments are among the most prevalent and functionally limiting challenges, with a substantial proportion of children with CP experiencing significant difficulties in speech production and communication (3).

Language development in children with CP is typically characterised by delays in both receptive and expressive domains compared to typically developing peers. Emerging evidence suggests that these impairments, particularly in expressive language, cannot be fully explained by motor speech limitations alone (4, 5). This indicates that underlying mechanisms of language impairment in CP extend beyond motor dysfunction and may involve broader neurophysiological processes.

Auditory processing constitutes a fundamental component of language development. It encompasses central nervous system mechanisms responsible for the perception, discrimination, and interpretation of speech sounds, forming the basis of language acquisition (6). Recent research has highlighted that deficits in auditory processing, particularly disruptions in neural timing mechanisms, are closely associated with delayed language development (7).

Cortical auditory evoked potentials (CAEPs) provide an objective method for assessing different stages of auditory cortical processing. Key components such as P1, N1, and mismatch negativity (MMN) reflect early sensory encoding, cortical processing, and automatic auditory discrimination, respectively (8). Prolonged latencies and reduced amplitudes in these components have been associated with delayed neural processing and reduced efficiency, and have been linked to language impairments in various developmental conditions (9).

Despite growing evidence on the relationship between auditory processing and language development in neurodevelopmental disorders, studies examining this relationship at the cortical level in children with CP remain limited. Existing research has largely focused on motor speech impairments or peripheral hearing, while the role of central auditory processing in language outcomes has not been sufficiently elucidated (2). Furthermore, the extent to which motor severity interacts with neurophysiological measures to influence language performance remains unclear (3). Early identification of cortical auditory processing deficits may have important clinical implications for children with cerebral palsy. Detecting these deficits at an early stage may facilitate timely implementation of targeted auditory and language interventions, potentially improving communication outcomes and supporting more individualized rehabilitation planning.

Therefore, the present study aimed to examine cortical auditory processing in children with CP using CAEP measures and to investigate its association with receptive and expressive language performance. In addition, the contribution of motor severity, as indexed by the Gross Motor Function Classification System (GMFCS), was evaluated. By integrating electrophysiological and behavioural measures, this study seeks to address a critical gap in the literature and to provide insight into the neurophysiological mechanisms underlying language difficulties in CP, with potential implications for clinical assessment.

## Hypotheses

**H1.** Children with CP will exhibit prolonged CAEP latencies (P1, N1, MMN) and reduced amplitudes compared to typically developing peers.

**H2.** Children with CP will demonstrate significantly lower receptive and expressive language scores than controls.

**H3.** Within the CP group, CAEP latencies will be negatively associated with language performance, whereas amplitudes will be positively associated with language outcomes.

**H4.** Increasing motor severity (GMFCS level) will be associated with prolonged CAEP latencies and reduced language performance.

**H5.** CAEP measures (particularly N1 latency) and GMFCS level will remain significantly associated with language performance after adjustment for age, hearing status, and cognitive status.

## Methods

### Study design and ethic

This study was designed as a cross-sectional case–control investigation to examine the relationship between cortical auditory processing and language development in children with cerebral palsy (CP). The study was conducted in a private rehabilitation centre in Malatya, Türkiye. Participant recruitment and data collection were conducted between 24 March 2026 and 31 May 2026. Participants

A total of 96 children aged between 5 and 12 years were included in the study. Participants were divided into two groups:

- CP group: Children with a clinical diagnosis of cerebral palsy
- Control group: Age- and gender-matched typically developing children

### Inclusion criteria

- Age between 5 and 12 years
- Clinical diagnosis of CP (for CP group)
- No neurological or developmental disorders (for control group)
- Sufficient cognitive ability to understand test instructions
- Adequate hearing to complete behavioral language assessment and electrophysiological recordings, including children with normal hearing or mild hearing loss (pure-tone average ≤40 dB HL).

### Exclusion criteria

- Moderate or greater hearing loss (pure-tone average>40 dB HL), active middle-ear pathology, or any condition preventing reliable electrophysiological recording.
- Genetic syndromes or neurodegenerative disorders
- Severe intellectual disability
- Active neurological conditions that could affect EEG recordings

Participants in the CP group were recruited from a private rehabilitation centre in Malatya, Türkiye. Eligible children with a clinical diagnosis of cerebral palsy who were attending the centre for routine rehabilitation sessions were invited to participate during their scheduled therapy visits. The control group was recruited from mainstream schools in the same geographic region. Typically developing children were invited to participate during scheduled school hours. Participation in both groups was entirely voluntary, and only children who met all predefined eligibility criteria were enrolled in the study.

Children with mild hearing loss (pure-tone average 26–40 dB HL) were eligible for inclusion because hearing status remained sufficient for reliable behavioral language assessment and cortical auditory evoked potential recording. Hearing status (normal hearing vs. mild hearing loss) was subsequently entered as a categorical covariate in all adjusted analyses.

### Language assessment

Receptive and expressive language skills were assessed using the Turkish Early Language Development Test (TEDİL), a standardized language assessment developed and validated for Turkish-speaking children. The Turkish version of TEDİL has demonstrated satisfactory psychometric properties, including validity and reliability, for assessing early language development in Turkish children (10).

The total language score was calculated as the mean of the receptive and expressive language scores. Because the total score was directly derived from these two component scores, very high intercorrelations among receptive, expressive, and total language scores were expected. To improve transparency, these intercorrelations are presented in Supplementary Table S1.

### Cortical auditory evoked potentials (CAEP) recording

#### EEG recording and preprocessing

EEG data were recorded using a 32-channel EEG system (Neurosoft, Russia) with Ag/AgCl sintered electrodes positioned according to the International 10–20 system. Signals were continuously sampled at 1,000 Hz and band-pass filtered online between 0.1 and 100 Hz. All recordings were referenced online to the linked mastoid electrodes, and electrode impedances were maintained below 5 kΩ throughout data acquisition.

Ocular and movement-related artifacts were identified and removed using a combination of automatic amplitude-based artifact rejection procedures and visual inspection. EEG epochs were segmented from −100 ms to 500 ms relative to stimulus onset and baseline-corrected using the −100 to 0 ms pre-stimulus interval. Trials exceeding ±100 μV at any electrode were excluded from further analysis. Artifact-free epochs were averaged separately for each participant.

ERP components were analyzed at the Cz and Fz electrode sites because of their established sensitivity to cortical auditory responses. Peak latency (ms) and peak amplitude (μV) were extracted using semi-automated peak detection supported by visual inspection.

#### Auditory stimulation

For the P1–N1 paradigm, auditory stimuli consisted of 1,000 Hz tone bursts (100 ms duration; 10 ms rise/fall time and 80 ms plateau) presented binaurally through headphones at 65 dB HL. Stimuli were delivered at a rate of 1 Hz (inter-stimulus interval = 1,000 ms). A total of 250 stimuli were presented, with at least 200 artifact-free epochs retained for averaging.

For the MMN paradigm, a passive auditory oddball design was employed. Standard stimuli consisted of 1,000 Hz tone bursts (80%; 400 trials), whereas deviant stimuli consisted of 1,200 Hz tone bursts (20%; 100 trials). Stimulus duration was 100 ms, presentation intensity was 65 dB HL, and the inter-stimulus interval was 500 ms. MMN responses were calculated as difference waveforms (deviant ERP minus standard ERP).

#### ERP component identification

ERP components were identified within predefined latency windows based on previous literature:

- **P1:** 50–80 ms (maximum positive peak)
- **N1:** 80–120 ms (maximum negative peak)
- **MMN:** 100–250 ms (maximum negative peak in the difference waveform)

For each component, peak latency (ms) and peak amplitude (μV) were extracted for statistical analyses.

#### Clinical assessment

Gross motor function was classified using the Gross Motor Function Classification System (GMFCS), a five-level classification system that categorizes the severity of motor impairment in children with cerebral palsy, with Level I representing the highest level of motor function and Level V the most severe motor limitations. For statistical analyses, GMFCS levels were grouped as I–II, III, and IV–V to reflect clinically meaningful categories of motor impairment while ensuring adequate sample sizes within each subgroup.

#### Ethical considerations

The study protocol was approved by the İnönü University Non-Interventional Clinical Research Ethics Committee (Approval No: 2026/9784; Session No: 6; Date: 24 March 2026). The study was conducted in accordance with the Declaration of Helsinki. Written informed consent was obtained from the parents or legal guardians of all participants.

#### Statistical analysis

Statistical analyses were performed using IBM SPSS Statistics (Version 26).

- Normality was assessed using the Shapiro–Wilk test
- Between-group comparisons were conducted using independent-samples *t*-tests or Mann–Whitney *U*-tests where appropriate
- Effect sizes were calculated using Cohen's d
- Associations between variables were examined using Pearson or Spearman correlation analyses
- Multiple linear regression analysis was performed to identify variables that remained statistically associated with language performance after simultaneous adjustment for the other variables included in the model.
- Analysis of covariance (ANCOVA) was used to control for potential confounding variables, including socioeconomic factors

To control for multiple comparisons, a false discovery rate (FDR) correction (Benjamini–Hochberg procedure) was applied where appropriate. Statistical significance was set at *p* < .05.

#### Power analysis

Sample size was determined using G*Power 3.1 software. Based on anindependent-samples *t*-test model with an alpha level of 0.05, power (1−*β*) of 0.80, and a large effect size (Cohen's d = 0.80), a minimum of 26 participants per group was required.

The final sample included 48 participants in each group, indicating that the study was adequately powered. The achieved statistical power for the primary comparisons was approximately 0.97.

## Results

### Participant characteristics

The final sample comprised 96 children: 48 with cerebral palsy (CP) and 48 age- and gender-matched typically developing controls (Table 1). The groups did not differ significantly in age (t = −0.16, *p* = .876) or gender distribution (*χ*^2^ = 0.70, *p* = .403), confirming successful matching.

**Table 1:** Demographic and clinical characteristics of participants.

Variable	Control (*n* = 48)	CP (*n* = 48)	Statistic	*P*
Age, years (M ± SD)	8.67 ± 1.93	8.73 ± 1.89	*t* = −0.16	0.876
Age range (min–max)	5.2–11.9	5.3–11.9	—	—
Gender, male/female	28/20	32/16	*χ*^2^ = 0.70	0.403
Hearing status, dB (M ± SD)	12.1 ± 2.8	18.4 ± 5.1	*t* = −7.45	<0.001
Hearing status — normal/mild loss	48/0	37/11	χ^2^ = 12.00	<0.001
Maternal education, years (M ± SD)	12.4 ± 2.8	10.6 ± 3.1	*t* = 3.02	0.004
Paternal education, years (M ± SD)	12.6 ± 2.7	10.9 ± 3.0	*t* = 2.89	0.005
Household income, TL (M ± SD)	36,300 ± 11,800	26,700 ± 10,200	*t* = 4.27	<0.001
Cognitive status — typical/mild delay	48/0	26/22	χ^2^ = 26.09	<0.001
CP type: Spastic/Dyskinetic/Ataxic	—	34/9/5	—	—
GMFCS I–II/III/IV–V	—	19/12/17	—	—
Epilepsy	—	9 (18.8%)	—	—
Visual impairment	—	4 (8.3%)	—	—

M ± SD or *n* (%). Group comparisons: independent-samples *t*-test (continuous) and chi-square (categorical). GMFCS, Gross Motor Function Classification System. Hearing loss defined as pure-tone average >25 dB HL.

Hearing status were significantly higher in the CP group (18.4 ± 5.1 dB) compared to controls (12.1 ± 2.8 dB; *t* = −7.45, *p* < .001). Although all values remained within clinically acceptable limits for electrophysiological assessment, hearing status was included as a covariate in sensitivity analyses to minimise potential peripheral auditory confounding. Including hearing status as a covariate did not materially change the pattern or significance of the results.

Socioeconomic variables differed significantly between groups. The CP group had lower maternal (*p* = .004) and paternal education (*p* = .005), as well as lower household income (*p* < .001). These variables were therefore included as covariates in subsequent analyses.

Within the CP group, 34 children (70.8%) had spastic CP, 9 (18.8%) dyskinetic CP, and 5 (10.4%) ataxic CP. Mild cognitive delay was present in 22 participants (45.8%). Comorbidities included epilepsy (18.8%) and visual impairment (8.3%).

### Between-group comparisons of CAEP components and language scores

**Figure 1:**
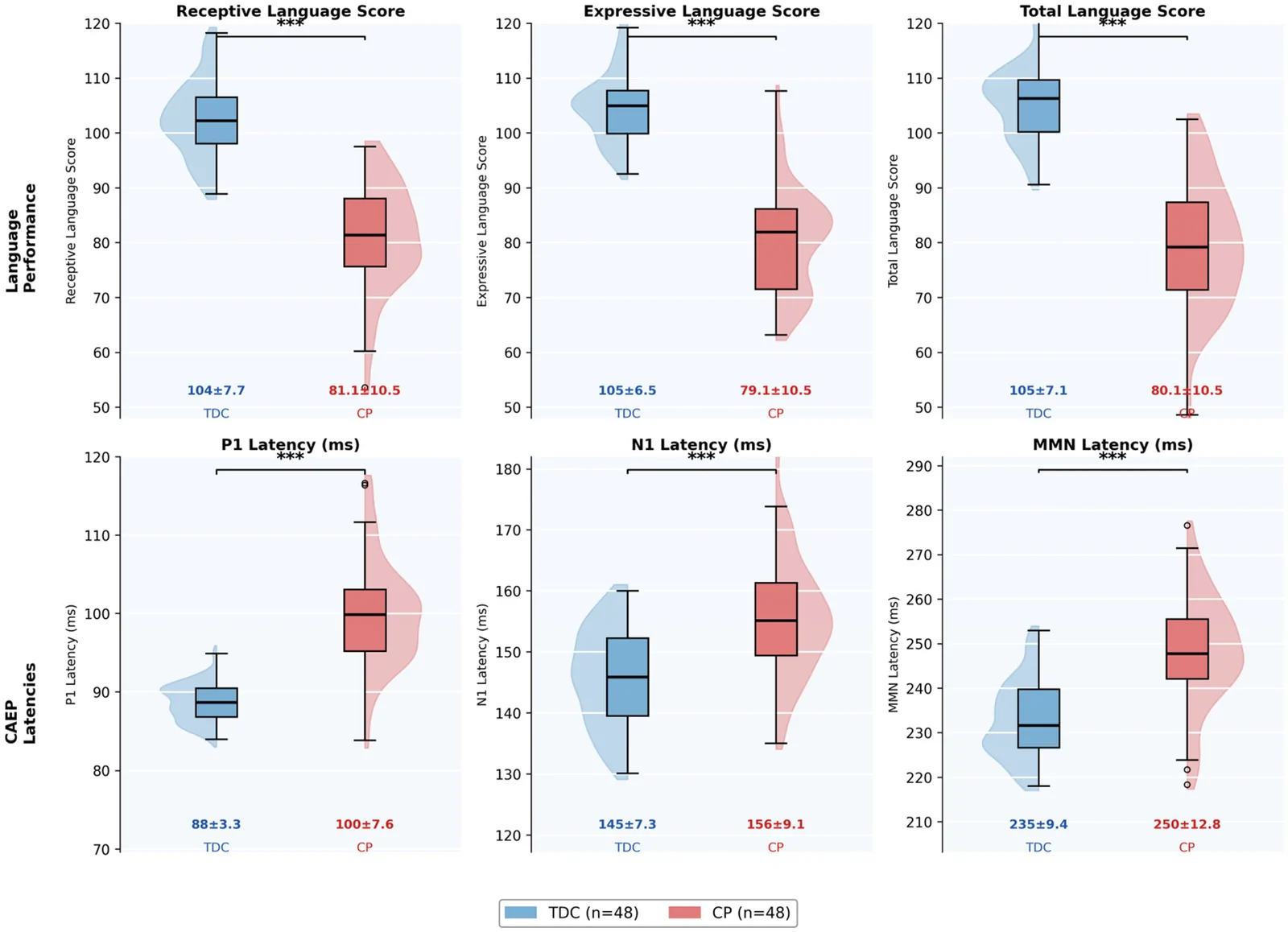
Comparison of language performance (top row) and CAEP latencies (bottom row) between CP and TDC groups. Violin plots represent data distribution; boxes indicate interquartile range; horizontal lines indicate median. Values below each plot show group mean ± standard deviation. *** *p* < .001.

Independent-samples *t*-tests revealed statistically significant differences between groups across all CAEP and language measures (Table 2, Figure 1).

**Table 2:** CAEP component and language score comparisons between CP and control groups.

Measure	Control M ± SD	CP M ± SD	t (94)	*P*	95% CI of *Δ*	*d*
CAEP Latencies						
P1 latency (ms)	88.41 ± 5.12	100.18 ± 7.24	9.21	<0.001	[9.19, 14.35]	1.88
N1 latency (ms)	142.28 ± 6.76	157.23 ± 8.91	9.15	<0.001	[11.74, 18.16]	1.87
MMN latency (ms)	234.13 ± 9.19	251.23 ± 12.54	7.61	<0.001	[12.67, 21.53]	1.55
CAEP Amplitudes						
P1 amplitude (µV)	2.66 ± 0.35	2.04 ± 0.38	8.39	<0.001	[−0.77, −0.47]	1.71
N1 amplitude (µV)	−5.26 ± 0.45	−3.94 ± 0.61	12.07	<0.001	[1.10, 1.54]	1.56
MMN amplitude (µV)	−3.11 ± 0.37	−2.03 ± 0.56	11.18	<0.001	[0.88, 1.28]	1.52
Standardised Language Scores						
Receptive language (SS)	105.83 ± 8.23	81.02 ± 10.19	13.06	<0.001	[−28.54, −21.08]	1.76
Expressive language (SS)	104.92 ± 7.58	79.06 ± 10.48	13.56	<0.001	[−29.72, −22.00]	1.57
Total language (SS)	105.38 ± 7.83	80.04 ± 10.37	13.47	<0.001	[−29.09, −21.59]	1.75

*Δ* = CP mean–Control mean. 95% CI = confidence interval of the mean difference. d = Cohen's d. SS = standard score (population M = 100, SD = 15). All tests two-tailed, df = 94.

**Figure 2:**
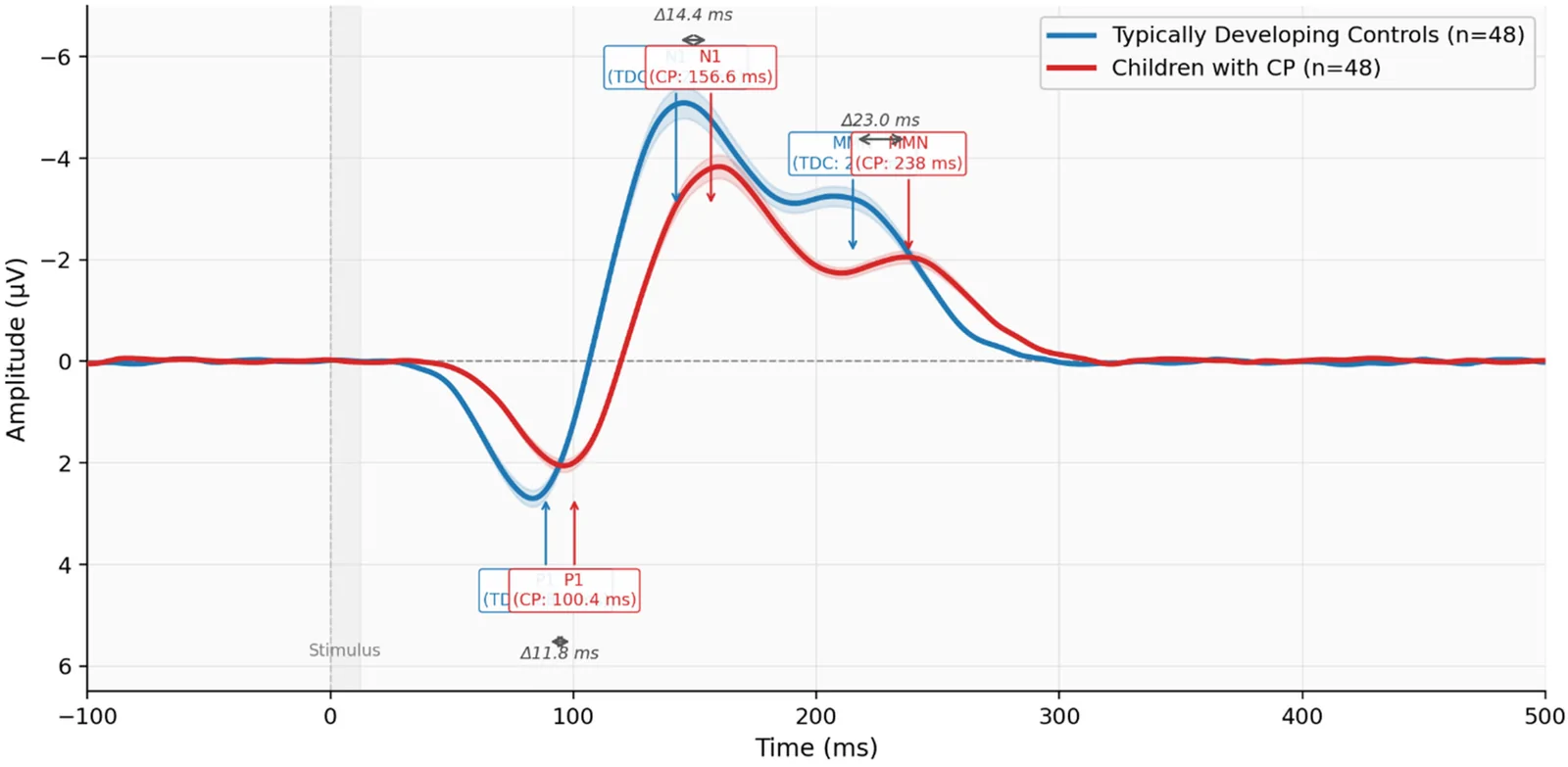
Grand-average cortical auditory evoked potential (CAEP) waveforms for children with cerebral palsy (CP, *n* = 48) and typically developing controls (TDC, *n* = 48). Shaded areas represent ±1 standard error of the mean (SEM). *Δ* indicates the latency difference between groups for each component (P1, N1, MMN). Negative polarity is plotted upward per ERP convention. All comparisons were statistically significant at *p* < .001.

Children with CP exhibited prolonged CAEP latencies, as illustrated in the grand-average waveforms (Figure 2):

- P1 latency: +11.77 ms (*d* = 1.88)
- N1 latency: +14.95 ms (*d* = 1.87)
- MMN latency: +17.10 ms (*d* = 1.55)

Amplitude values were significantly reduced:

- P1 amplitude: −0.62 µV (*d* = 1.71)
- N1 amplitude: −1.32 µV (*d* = 1.56)
- MMN amplitude: −1.08 µV (*d* = 1.52)

Language scores were markedly lower in the CP group:

- Receptive language: *d* = 1.76
- Expressive language: *d* = 1.57
- Total language: *d* = 1.75

All comparisons were statistically significant at *p* < .001.

To control for multiple comparisons, a false discovery rate (FDR) correction (Benjamini–Hochberg procedure) was applied, and all results remained statistically significant after correction.

To further account for potential confounding, additional ANCOVA analyses were conducted including hearing status and cognitive status as covariates. Group differences in CAEP measures and language scores remained statistically significant after adjustment (all *p* < .001), indicating that these differences cannot be fully explained by peripheral hearing or cognitive factors. The large effect sizes observed may reflect the clinical severity and heterogeneity within the CP sample.

### Associations between CAEP measures and language scores

Within the CP group, Pearson correlation analyses revealed significant linear associations between CAEP measures and language performance (Table 3). N1 latency demonstrated the strongest negative correlations with receptive language (*r* = −0.989, *p* < .001), expressive language (*r* = −0.989, *p* < .001), and total language scores (*r* = −0.989, *p* < .001). Similarly, P1 latency (*r* = −0.994) and MMN latency (*r* = −0.978) were also strongly negatively correlated with all language measures (all *p* < .001). MMN amplitude was likewise strongly associated with language performance (*r* = −0.995, *p* < .001). Because MMN amplitudes were recorded as negative-going voltages, this negative correlation indicates that larger (i.e., more negative) MMN responses were associated with better language performance. After false discovery rate (FDR) correction, all reported correlations remained statistically significant. No significant correlations were observed in the control group (all *p* > .10), whereas significant associations were observed in the CP group.

**Table 3:** Pearson correlations between CAEP measures and language scores in children with cerebral palsy (CP) (*n* = 48).

CAEP Measure	Receptive *r*	Expressive *r*	Total *r*	*P* (all)
P1 latency	−0.994	−0.994	−0.994	<0.001
N1 latency	−0.989	−0.989	−0.989	<0.001
MMN latency	−0.978	−0.978	−0.978	<0.001
MMN amplitude	−0.995	−0.995	−0.995	<0.001

Pearson correlation coefficients are presented for children with cerebral palsy (*n* = 48). Negative coefficients indicate that longer CAEP latencies were associated with lower language scores. For MMN amplitude, more negative amplitudes indicate larger cortical responses. All reported correlations were statistically significant (*p* < .001).

### Within-CP subgroup analyses

Tables 4, 5 presents subgroup comparisons within the CP group by CP type (Panel A), GMFCS level (Panel B), and cognitive status (Panel C). All post-hoc comparisons were Bonferroni- corrected.

**Table 4:** CAEP and language measures across CP type, GMFCS level, and cognitive status.

Measure	Group 1	Group 2	Group 3	F/t	*P*	Post-hoc/ES
Panel A. CP Type — One-Way ANOVA: Spastic (*n* = 34) | Dyskinetic (*n* = 9) | Ataxic (*n* = 5)						
P1 latency (ms)	98.52 ± 5.78	111.23 ± 4.91	98.82 ± 2.18	F = 14.27	<0.001	D > S, A
N1 latency (ms)	155.08 ± 6.44	172.82 ± 6.28	155.02 ± 3.48	F = 21.68	<0.001	D > S, A
MMN latency (ms)	248.29 ± 9.12	268.13 ± 8.44	248.16 ± 3.31	F = 17.91	<0.001	D > S, A
MMN amplitude (µV)	−2.23 ± 0.41	−1.31 ± 0.34	−2.10 ± 0.14	F = 19.86	<0.001	D < S, A
Total language (SS)	85.12 ± 6.88	66.00 ± 4.00	82.60 ± 1.52	F = 33.71	<0.001	S,A > D
Panel B. GMFCS Level — One-Way ANOVA: I–II (*n* = 19) | III (*n* = 12) | IV–V (*n* = 17)						
P1 latency (ms)	92.73 ± 2.75	102.92 ± 2.37	109.33 ± 4.07	F = 115.95	<0.001	I–II < III < IV–V
N1 latency (ms)	147.27 ± 3.32	158.96 ± 2.46	170.62 ± 5.12	F = 155.21	<0.001	I–II < III < IV–V
MMN amplitude (µV)	−2.62 ± 0.19	−2.02 ± 0.20	−1.35 ± 0.28	F = 137.00	<0.001	I–II > III > IV–V
Total language (SS)	91.45 ± 3.70	77.63 ± 3.51	68.26 ± 4.10	F = 161.72	<0.001	I–II > III > IV–V
Panel C. Cognitive Status — Independent *t*-test: Typical Cognition (*n* = 26) | Mild Delay (*n* = 22)						
P1 latency (ms)	95.72 ± 4.81	105.48 ± 6.14	—	t = 6.21	<0.001	d = 1.80
N1 latency (ms)	151.10 ± 5.67	164.38 ± 7.89	—	t = 6.80	<0.001	d = 1.97
MMN amplitude (µV)	−2.42 ± 0.42	−1.58 ± 0.38	—	t = 7.23	<0.001	d = 2.09
Total language (SS)	88.58 ± 4.89	70.00 ± 5.60	—	t = 12.31	<0.001	d = 1.76

Panel A: one-way ANOVA; Bonferroni *post-hoc* — D = dyskinetic, S = spastic, A = ataxic. Panel B: GMFCS grouped as I–II (mild), III (moderate), IV–V (severe); one-way ANOVA. Panel C: independent-samples *t*-test; d = Cohen's d. All *p*-values two-tailed.

**Table 5:** Multiple linear regression analysis of factors associated with total language scores in children with cerebral palsy (*n* = 48).

Predictor	B	SE	*β*	*t*	*p*
Age	−0.084	0.097	−0.015	−0.868	.390
Hearing status	−2.373	0.724	−0.095	−3.278	.002
GMFCS level	−2.702	0.685	−0.299	−3.943	<.001
Cognitive status	1.909	0.659	0.090	2.895	.006
N1 latency	−0.803	0.098	−0.700	−8.214	<.001

Model statistics: R^2^ = 0.987, Adjusted R^2^ = 0.986, F(5,42) = 662.10, *p* < .001. Because receptive, expressive, and total language scores were highly intercorrelated, only the total language score was entered into the regression model to avoid redundancy. Variance inflation factors indicated substantial multicollinearity between N1 latency and GMFCS level. Therefore, standardized regression coefficients should be interpreted with caution.

### CP type

Significant differences were observed across CP subtypes (all *p* < .001). The dyskinetic subgroup demonstrated the most severe profile, with the longest CAEP latencies, lowest MMN amplitudes, and lowest language scores. No significant differences were observed between spastic and ataxic subtypes.

### Motor severity (GMFCS)

A clear dose–response relationship was observed across GMFCS levels. Increasing motor severity was associated with progressively prolonged CAEP latencies, reduced amplitudes, and lower language scores (all *p* < .001). These relationships remained significant after controlling for cognitive status.

### Cognitive status

Children with mild cognitive delay exhibited significantly prolonged CAEP latencies, reduced MMN amplitudes, and lower language scores compared with those with typical cognition (all *p* < .001). However, when cognitive status was included as a covariate, the associations between CAEP measures and language outcomes remained statistically significant, indicating that these associations persisted after adjustment for cognitive status (Table 6).

**Table 6:** Analysis of covariance (ANCOVA) comparing total language scores between children with cerebral palsy and typically developing controls after adjustment for potential confounders.

Source	*F*	*p*
Group	112.08	<.001
Maternal education	0.06	.815
Household income	0.04	.853
Cognitive status	25.22	<.001
Hearing status	11.26	.001

Model statistics: R^2^ = .836; Adjusted R^2^ = .827.

### Multivariable regression analysis of language outcomes

A multiple linear regression analysis was performed to identify variables that remained statistically associated with total language scores in children with cerebral palsy after simultaneous adjustment for the other variables included in the model.

The model included N1 latency, GMFCS level, cognitive status, age, and hearing status and was statistically significant, explaining 98.7% of the variance in language performance (R^2^ = .987, adjusted R^2^ = .986, F(5,42) = 662.10, *p* < .001).

Significant independent variables:

- N1 latency (*β* = −.700, *p* < .001)
- GMFCS level (*β* = −.299, *p* < .001)
- Hearing status (*β* = −.095, *p* = .002)
- Cognitive status (*β* = .090, *p* = .006)

Non-significant variable:

- Age (*β* = −.015, *p* = .390)

These findings indicate that N1 latency and GMFCS level both remained statistically associated with language performance when entered into the same multivariable regression model. However, because substantial multicollinearity was present between these predictors, their individual regression coefficients should be interpreted with caution. Higher GMFCS levels and poorer hearing status were associated with lower language scores, whereas better cognitive status was associated with higher language performance. Age was not significantly associated with language performance after adjustment for the other variables.

### Adjusted group comparisons (ANCOVA)

Analysis of covariance (ANCOVA) was performed to compare total language scores between children with cerebral palsy and typically developing controls after adjustment for maternal education, household income, cognitive status, and hearing status. The overall model was statistically significant (R^2^ = .836, adjusted R^2^ = .827, *p* < .001). Group remained significantly associated with language performance after adjustment for the covariates (F(1,90) = 112.08, *p* < .001). Cognitive status (*F* = 25.22, *p* < .001) and hearing status (*F* = 11.26, *p* = .001) were also significantly associated with language scores, whereas maternal education (*p* = .815) and household income (*p* = .853) were not significant covariates.

### Gender effects within the CP group

No significant gender differences were observed within the CP group across any CAEP or language measures (all *p* > .70), and gender was not included in further analyses.

### Summary

Children with CP demonstrated marked impairments in cortical auditory processing and language performance compared to typically developing peers. These differences remained robust after controlling for cognitive, socioeconomic, and auditory factors.

Within the CP group, variability in outcomes was associated with CP subtype, motor severity, and cognitive status.

Multiple regression analyses showed that N1 latency and GMFCS level both remained statistically associated with language performance when entered into the same multivariable model. The final model explained 98.7% of the variance in total language scores (R^2^ = .987), with N1 latency showing the strongest statistical association with language performance.

Overall, these findings indicate that cortical auditory processing remained strongly associated with language outcomes in children with CP after adjustment for motor severity, cognitive status, age, and hearing status.

## Discussion

The present study examined the relationship between cortical auditory processing and language performance in children with cerebral palsy (CP), integrating electrophysiological, motor, and cognitive dimensions within a unified analytical framework. The findings indicate that children with CP exhibit marked alterations in cortical auditory evoked potentials (CAEPs), alongside substantially lower receptive and expressive language performance compared with typically developing peers. Importantly, these differences remained robust after adjustment for socioeconomic variables, hearing status, and cognitive status, supporting an association between central auditory processing and language outcomes in this population.

Consistent with our hypotheses, children with CP demonstrated prolonged latencies and reduced amplitudes across the evaluated CAEP components. These findings indicate delayed and less efficient cortical auditory processing, in line with developmental models that conceptualize CAEP latencies as indices of neural maturation and temporal processing efficiency (6). In particular, prolonged N1 and MMN latencies may reflect disruptions in cortical timing mechanisms that are essential for speech perception and language acquisition. Previous research has shown that cortical auditory responses are closely linked to auditory threshold estimation and neural processing efficiency, further supporting their relevance for higher-order auditory processing (11).

Language outcomes in the CP group were substantially lower than those of typically developing children, supporting previous evidence that language impairments in CP extend beyond motor speech limitations (3, 5). Similarly, research on speech and language development in CP highlights the heterogeneity of communication impairments and the contribution of multiple underlying mechanisms beyond motor dysfunction (3). The magnitude of the differences observed in the present study underscores the multifactorial nature of language difficulties in CP, involving not only motor constraints but also central auditory and cognitive processes.

One of the principal findings of the present study is the identification of systematic associations between CAEP parameters and language performance. Prolonged CAEP latencies were negatively associated with language scores, whereas MMN amplitude showed a positive relationship with language outcomes. These findings are consistent with prior work demonstrating that mismatch negativity (MMN) reflects automatic auditory discrimination processes and serves as a sensitive indicator of central auditory processing efficiency in both typical and clinical populations (8). Moreover, MMN has been widely used to investigate auditory processing deficits in developmental language disorders, supporting its relevance for language-related neural mechanisms (9). Although these associations were not observed in the typically developing control group, this finding should be interpreted with caution. The relatively restricted range of language performance and CAEP measures among typically developing children may have limited the ability to detect significant correlations. Therefore, the absence of significant associations in the control group should not be interpreted as evidence that the relationship between cortical auditory processing and language is unique to children with cerebral palsy. Rather, the greater variability in both language abilities and neurophysiological measures within the CP group may have made these associations more readily detectable. Although the observed correlations were exceptionally strong, these findings should be interpreted within the context of the relatively homogeneous clinical characteristics of the CP sample and the cross-sectional design, which precludes causal inference. Future studies using larger, multicentre, independent cohorts are needed to confirm the reproducibility and generalizability of these findings. Accordingly, these associations should not be interpreted as evidence that cortical auditory processing directly determines language performance but rather as indicating a close relationship between these variables within this study population.

Among the electrophysiological measures, N1 latency remained the strongest statistically significant variable in the multivariable regression model. These findings suggest that N1 latency may provide complementary information regarding language performance in children with cerebral palsy, although the cross-sectional design precludes conclusions regarding causality. These findings suggest that higher-order cortical auditory processing may contribute to language performance in children with cerebral palsy. N1 latency has been associated with auditory discrimination, neural timing, and cortical maturation processes and has been shown to vary as a function of auditory development and sensory experience (12). Given that cortical auditory responses continue to mature throughout childhood, age-related developmental changes may also have contributed to variability in CAEP measures. However, age was included as a covariate in the regression analyses, and the association between N1 latency and language performance remained significant after adjustment, suggesting that the observed relationship was not solely attributable to developmental maturation. Age was not significantly associated with language performance in the final regression model after adjustment for the other variables. In addition, studies in pediatric populations have demonstrated that earlier cortical responses, including P1 latency, are related to language outcomes and auditory development (13), supporting the broader relevance of cortical auditory markers in language acquisition. Importantly, the association between N1 latency and language performance in the present study remained significant after accounting for motor severity, cognitive status, and hearing status, indicating that this relationship cannot be explained solely by these factors. Similarly, although hearing status was retained as a significant covariate in the final regression model, the persistence of the association between N1 latency and language performance after adjustment suggests that the observed relationship cannot be explained solely by peripheral hearing function. Nevertheless, these findings should be interpreted in the context of the clinical heterogeneity of cerebral palsy. The CP group included children with different clinical subtypes, varying levels of motor impairment, and comorbid conditions such as mild cognitive delay, epilepsy, and visual impairment, all of which may have influenced both cortical auditory responses and language performance. Therefore, N1 latency should be considered a potentially useful complementary marker rather than a standalone indicator of language-related auditory processing in children with cerebral palsy. The exceptionally high proportion of explained variance and the presence of multicollinearity between N1 latency and GMFCS level suggest that these variables share substantial overlapping information within this relatively homogeneous clinical sample. Consequently, although N1 latency remained statistically associated with language performance in the multivariable model, its regression coefficient should be interpreted with caution because substantial multicollinearity indicated considerable shared variance with GMFCS level. Future studies using larger, multicentre, and independent cohorts are warranted to determine whether these findings can be replicated.

Motor severity demonstrated a clear dose–response relationship with both electrophysiological and language measures. In the multivariable model, both GMFCS level and N1 latency remained statistically associated with language performance after simultaneous adjustment for age, hearing status, and cognitive status. However, because these predictors exhibited substantial shared variance, their individual regression coefficients should be interpreted with caution. However, in the multivariable regression model, both GMFCS level and N1 latency remained statistically significant after simultaneous adjustment for age, hearing status, and cognitive status, indicating that motor impairment alone does not fully account for language outcomes in CP. Similarly, although cognitive status contributed to variability, it did not fully explain the observed relationships. These findings support a multidimensional model of language development, in which auditory, motor, and cognitive systems interact. Taken together, the present findings suggest that cortical auditory processing provides complementary information beyond motor severity and conventional clinical assessment. If confirmed in larger longitudinal studies, CAEP measures may contribute to the early identification and monitoring of language difficulties in children with cerebral palsy. This interpretation is also consistent with longitudinal findings showing that language comprehension and functional communication in CP follow diverse developmental trajectories influenced by multiple interacting factors (14). In addition, the subgroup analyses according to CP subtype and GMFCS level should be interpreted with caution. Although these analyses provide clinically relevant descriptive information, some CP subtypes, particularly the dyskinetic and ataxic groups, included relatively small numbers of participants, which may have limited statistical power. Therefore, these findings should be regarded as exploratory and require confirmation in larger, more diverse cohorts.

## Strengths and limitations

This study has several strengths. First, it integrates electrophysiological, behavioural, and clinical variables within a single analytical framework, allowing for a comprehensive evaluation of language outcomes in CP. Second, the use of objective CAEP measures provides insight into underlying neural mechanisms beyond behavioural assessment. Third, the inclusion of multiple covariates, including socioeconomic factors, cognitive status, and hearing status, strengthens the validity of the findings.

However, several limitations should be acknowledged. The cross-sectional design precludes causal inferences regarding the directionality of the observed associations. Although the sample size was adequate for primary analyses, subgroup analyses were based on relatively small numbers and should be interpreted with caution. Additionally, the study was conducted in a single clinical setting, which may limit generalisability.

In addition, the cross-sectional design precluded longitudinal follow-up, limiting our ability to evaluate changes in cortical auditory processing and language performance over time. Furthermore, although participants ranged in age from 5 to 12 years, the present study was not designed to examine age-related developmental changes within this age range. Future longitudinal studies with repeated assessments are needed to clarify developmental trajectories and the temporal relationship between cortical auditory processing and language outcomes in children with cerebral palsy.

Furthermore, the relatively broad age range of the participants (5–12 years) may have introduced developmental variability in cortical auditory responses despite statistical adjustment for age. The clinical heterogeneity of the cerebral palsy group, including differences in CP subtype, motor severity, and comorbid conditions such as epilepsy and visual impairment, may also have influenced both electrophysiological and language measures. Finally, although multiple potential confounders were considered in the analyses, it remains difficult to fully disentangle the contribution of central auditory processing from overall neurodevelopmental severity in children with cerebral palsy. Future longitudinal studies with larger and more homogeneous cohorts are warranted to further clarify these complex relationships.

## Conclusion

In conclusion, the present study demonstrates that cortical auditory processing remained strongly associated with language performance in children with cerebral palsy after adjustment for motor severity, cognitive status, age, and hearing status. These findings support the potential value of CAEP measures as complementary neurophysiological markers in the assessment of language impairment in this population. These findings highlight the importance of central auditory mechanisms in understanding language difficulties in CP and support the potential role of electrophysiological measures as complementary tools in clinical assessment.

## Data Availability

The raw data supporting the conclusions of this article will be made available by the authors, without undue reservation.
