# Supplementary material for: Language performance in children with cerebral palsy: the role of cortical auditory processing beyond motor and cognitive factors
Source: Front Pediatr. 2026 Aug 31;14:1908510. doi: 10.3389/fped.2026.1908510 (PMC13572691; doi:10.3389/fped.2026.1908510)
Supplement: Supplementary file 1 [file Table1.docx]

**Supplementary Table S1**

**Supplementary Table S1.** Pearson correlations among receptive, expressive, and total language scores in the cerebral palsy group (n = 48).

| Variable | Receptive language | Expressive language | Total language |
| --- | --- | --- | --- |
| Receptive language | 1.000 | 0.9999** | 0.99998** |
| Expressive language | 0.9999** | 1.000 | 0.99998** |
| Total language | 0.99998** | 0.99998** | 1.000 |

**Note.** Pearson correlation coefficients. **p < .001** for all pairwise correlations. The total language score was calculated as the mean of the receptive and expressive language scores; therefore, very high intercorrelations among these variables were expected.
